# Role of Pten in leukemia stem cells

**DOI:** 10.18632/oncotarget.119

**Published:** 2010-06-07

**Authors:** Cong Peng, Yaoyu Chen, Dongguang Li, Shaoguang Li

**Affiliations:** ^1^Division of Hematology/Oncology, Department of Medicine, University of Massachusetts Medical School, 364 Plantation Street, Worcester, MA 01605, USA; ^2^School of Computer and Security Science, Edith Cowan University, 2 Bradford Street, Mount Lawley, WA 6050, Australia

**Keywords:** Pten, BCR-ABL, leukemic stem cells, CML, therapeutic potential

## Abstract

Chronic myeloid leukemia (CML) is initiated from the BCR-ABL-expressing leukemia stem cells (LSCs). These LSCs are highly resistant to BCR-ABL kinase inhibitors, imatinib, dasantinib and nilotinib, and methods for eradication of LSCs are still not available. It is critical to identify genes that play roles in survival and proliferation of LSCs. We recently discovered that the tumor suppressor gene Pten is downregulated in LSCs of CML mice. By genetic deletion or overexpression of Pten, we confirmed that Pten functions as a tumor suppressor in LSCs of CML, consistent with the role of Pten in LSCs of acute myeloid leukemia (AML) and progenitor cells of T-ALL progenitors. Functional enhancement of the Pten pathway provides a therapeutic strategy for targeting LSCs.

## INTRODUCTION

The human Philadelphia chromosome (Ph) is present in over 95% of CML cases [1], and arises from a reciprocal translocation between chromosome 9 and 22, resulting in the formation of the chimeric BCR-ABL oncogene. BCR-ABL encodes a constitutively activated, oncogenic tyrosine kinase [2]. The BCR-ABL kinase inhibitor imatinib induces a complete hematologic and cytogenetic response in the majority of CML patients [3], but is unable to completely eradicate BCR-ABL-expressing leukemic cells, suggesting that LSCs are not eliminated. Over time, patients can become drug resistant and develop progressive disease despite continued treatment [4-6].

LSCs in many types of hematologic malignancies are believed to be a cell population required for initiating and sustaining growth of the leukemia [7-13]. In CML patients, bone marrow CD34+Lin- cells, in which normal hematopoietic stem cells (HSCs) reside, are thought to contain CML stem cells and be responsible for disease initiation, progression and resistance to imatinib [14, 15]. Several clinical reports have confirmed that disease relapse occurred in CML patients who had achieved complete molecular response after imtainib treatment but discontinued the therapy [16, 17]. These results indicate that for some reasons, LSCs find ways to survive the treatment by BCR-ABL kinase inhibitors.

### LSCs in CML are insensitive to inhibition by BCR-ABL kinase inhibitors

A study showed that a quiescent cell population in LSCs (Lin-CD34+) from CML patients was resistant to imatinib [14], suggesting that LSCs are insensitive to a BCR-ABL kinase inhibitor. To support this idea, two second-generation BCR-ABL kinase inhibitors, dasatinib and nilotinib, were used to treat LSCs in vitro. Dasatinib is a dual Src/BCR-ABL kinase inhibitor and exhibits a much greater potency than imatinib [18]. Although dasatinib led to significant inhibition of BCR-ABL kinase activity, the most primitive quiescent CML LSCs (Lin-CD34+CD38-) were resistant to dasatinib treatment [19]. Similarly, nilotinib with a potency which is 20-fold higher than imatinib could not induce apoptosis of LSCs [20]. In CML mice treated with imatinib or dasatinib, we showed that although these two drugs dramatically prolonged survival of CML mice, all diseased mice eventually died of CML due to the failure of imatinib and dasatinib to eradicate LSCs [21]. New genes with therapeutic potential are needed to be identified for targeting LSCs.

### Role of Pten in LSCs

To identify genes that are differentially regulated by BCR-ABL in LSCs, we compared the global gene expression between normal hematopoietic stem cells (HSCs) and LSCs by conducting a DNA microarray analysis [22]. We found that *Pten* is significantly downregulated by BCR-ABL [23]. Because *Pten* is often deleted or inactivated in many human cancers, including glioblastoma [24], endometrial carcinoma [25], and lymphoid malignancies [26], we decided to test whether Pten also functions as a tumor suppressor in survival of LSCs and CML development. We first assessed whether genetic deletion of *Pten* facilitates CML development by using *Pten* conditional knockout mice (*Pten*^fl/fl^) as donor mice in our retroviral transduction/transplantation mouse model of CML. In so doing, bone marrow cells of *Pten*^fl/fl^ mice were transduced with BCR-ABL-iCre-GFP retrovirus or BCR-ABL-GFP retrovirus (as a control), followed by transplantation of the transduced cells into lethal irradiated recipient mice. Mice receiving donor cells transduced with BCR-ABL-iCre-GFP developed CML much faster that mice receiving donor cells transduced with BCR-ABL-GFP, with a higher percentage of myeloid leukemia cells and more severe infiltration of leukemia cells in the lungs. To confirm the role of Pten in CML, we examined whether overexpression of Pten causes a delay of CML development. We induced CML in mice with BCR-ABL-Pten-GFP or BCR-ABL-GFP, and found that CML development was significantly slower when Pten was overexpressed. Pten overexpression caused cell cycle arrest and increased apoptosis of leukemia cells. Next, we examined whether *Pten* suppresses LSCs. To do so, we compared the percentages of LSCs with and without ectopically expressed Pten, and found that Pten overexpression caused a decrease in the percentage of bone marrow LSCs, suggesting that Pten has an inhibitory effect on LSCs. To support this observation, we sorted LSCs from mice with CML induced by BCR-ABL-Pten-GFP or BCR-ABL-GFP, followed by transplantation of these LSCs into secondary recipients. We found that Pten overexpression reduced the ability of LSCs to induce CML [27]. Because mTOR is hyperactived or upregulated when Pten is mutated or deteleted in human cancers [28], we treated LSCs from CML mice or human CML cell line K562 *in vitro* with rapamycin, a mTOR inhibitor, and found that rapamycin induced apoptosis of these cells, suggesting that a blockade of the mTOR pathway may help to inhibit LSCs and CML development.

### Potential mechanisms of Pten in LSCs

Although Pten is intensively studied in solid tumors and T cell-acute lymphoid leukemia (T-ALL) [29-32], little is known about Pten in CML until we show that Pten inhibits LSCs and CML development [27]. This result is supported by a clinical study which compared globe gene expression between normal CD34+ HSCs and CD34+ subsets from six patients with chronic phase CML. Besides the changes of gene expression for several adhesion molecules, transcription factors, cell cycle and stem cell fate regulators, Pten was also downregulated [33]. Another study showed that the gene expression profiles of mononuclear cells from CML patients who achieved complete cytogenetic response after imatinib treatment also indicated the Pten downregulation [34]. However, the mechanisms of Pten regulation of LSC function in CML need to be investigated. We noticed that the level of phosphorlated-Akt (p-Akt) was significantly lower in leukemia cells from CML mice when Pten was overexpressed [27], suggesting that p-Akt is a critical player of the Pten pathway. This idea is supported by our finding that induction of B-cell acute lymphoblastic leukemia (B-ALL) in mice was largely compromised when Akt1 was absent, as shown by the prolonged survival of recipients of BCR-ABL transduced Akt-deficient bone marrow cells mice [27]. The involvement of Akt1 in cancer has been shown in endometrium tumor, prostate cancer, thyroid tumor, adrenal medulla tumors and intestinal polyps in Pten+/- mice [35]. However, the roles of the Akt family members (Akt1, Akt2 and Akt3) in CML need to be studied further in the future. We have shown that expression of the Alox5 gene is upregulated by BCR-ABL in CML LSCs [36], and it has been reported that *Alox5* activates p-Akt through oxidation and inhibition of Pten [37]. The functional relationship between Pten and Alox5 needs to be studied.

When *Pten* is specifically deleted in mouse hematopoietic cells, the mice develop acute myeloid leukemia (AML) and acute lymphoid leukemia, and all mice died within 4 weeks [38, 39]. LSCs in these mice are highly enriched in Lin-Sca1+cKit+Flk2-CD48- population [38]. A blockade of differentiation from LT-HSC (Lin-Sca1+cKit+Flk2-) to ST-HSC (Lin-Sca1+cKit+Flk2+) was also found in *Pten* deficient mice, causing an eventual exhaustion of LT-HSC [39]. Increased percentage of S+G2M dividing HSCs was observed in *Pten* deficient mice, indicating that *Pten* functions as a molecular switch governing the G0-G1 transition between the quiescent and activated states of LT-HSCs to maintain normal HSCs pool [39]. The role of Pten in cell cycle control is consistent with our result that Pten expression induces cell cycle arrest in BCR-ABL expressing leukemia cells [27]. In addition, cyclin D1 is a well known target of the PI3K-Akt pathway [40], maintaining cells at G1 stage in preparation for G1/S phase transition. In *Pten* deficient AML mice, a high number of cyclin D1-expressing cells were detected in bone marrow, suggesting that cyclin D1 is downstream of Pten in cell cycle regulation of HSCs. Thus, the role of cyclin D1 in cell cycle regulation of LSCs in CML requires further investigation. Furthermore, after rapamycin administration, LSCs were depleted and normal HSCs restored in Pten-deficient AML mice [39], indicating that Pten maintains normal HSCs pool and suppresses LSCs through mTOR. It has been reported that PML (promyelocytic leukemia protein) plays a role in normal HSCs and BCR-ABL transduced quiescent LSCs, facilitating leukemia initiation and maintenance [41]. *Pml* deficiency promoted transition of LSCs from quiescent to activated stage and *Pml*-/- LSCs finally failed to initiate CML disease contrary to wild type LSCs after serial transplantation. As PML is a repressor of mTOR, inhibition of mTOR with rapamycin restored *Pml*-/- HSCs and the long-term reconstitution functions of LSCs. Another Pten conditional deletion mouse model demonstrated that Pten partial deletion in mouse fetal liver HSCs and their differentiated progeny led to a myeloidprliferative disorder, followed by actue T-lymphoblastic leukemia (T-ALL) [42]. In this study, the *Pten* deficient Lin-c-KitmidCD3+ population was shown to be the T-ALL LSCs through a serial transplantation assay. Interestingly, ablation of one allele of β-catenin significantly delayed the occurrence of acute leukemia. We and others have also shown that β-catenin plays a key role in maintaining LSCs in CML [43, 44] and AML [45].

**Figure 1.**
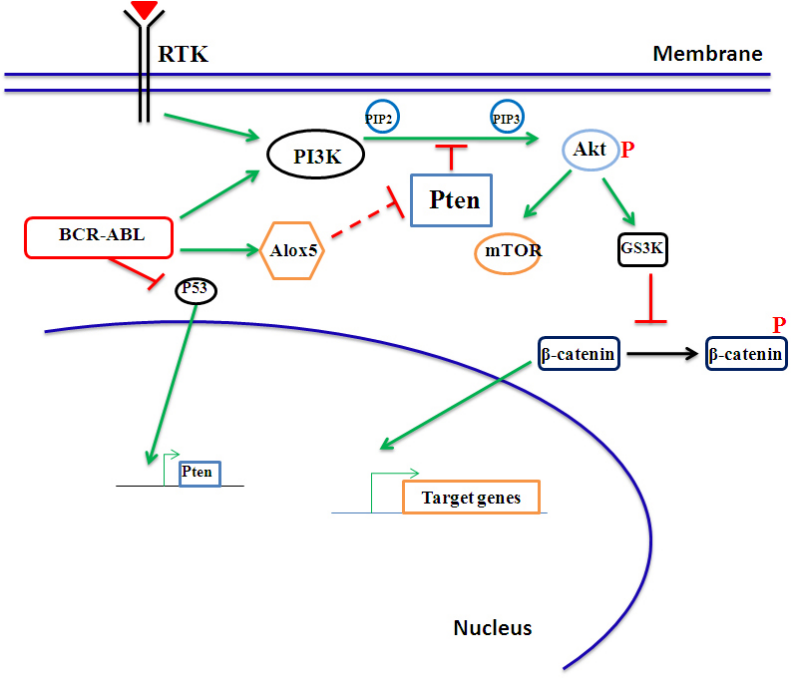
Molecular pathways in LSCs of CML In LSCs, BCR-ABL activates multiple signaling pathways that are normally activated by a receptor tyrosine kinase (RTK). In particular, the PI3K and Wnt/β-catenin pathways are critically involved. The novel Alox5 pathway we identified plays a specific role in LSCs but not normal HSCs (see the text for detail), and this pathway may interacts with the tumor suppressor gene Pten. The functional relationship between Alox5 and Pten is an important research topic that needs to be studied further.

Taken together, these results allow us to draw a picture connecting Pten with other key pathways involved in survival and proliferation of LSCs, including β-catenin, p53, Alox5, PI3K/Akt/mTOR pathways (Figure 1). These pathways are disturbed in CML, AML and other malignancies, and targeting of the pathways may be beneficial to patients.

## References

[1] Westbrook CA, Hooberman AL, Spino C, Dodge RK, Larson RA, Davey F, Wurster-Hill DH, Sobol RE, Schiffer C, Bloomfield CD (1992). Clinical significance of the BCR-ABL fusion gene in adult acute lymphoblastic leukemia: a Cancer and Leukemia Group B Study (8762). Blood.

[2] Wong S, Witte ON (2004). The BCR-ABL story: bench to bedside and back. Annu Rev Immunol.

[3] Druker BJ, Talpaz M, Resta DJ, Peng B, Buchdunger E, Ford JM, Lydon NB, Kantarjian H, Capdeville R, Ohno-Jones S, Sawyers CL (2001). Efficacy and safety of a specific inhibitor of the BCR-ABL tyrosine kinase in chronic myeloid leukemia. N Engl J Med.

[4] Weisberg E, Griffin JD (2000). Mechanism of resistance to the ABL tyrosine kinase inhibitor STI571 in BCR/ABL-transformed hematopoietic cell lines. Blood.

[5] Gorre ME, Mohammed M, Ellwood K, Hsu N, Paquette R, Rao PN, Sawyers CL (2001). Clinical resistance to STI-571 cancer therapy caused by BCR-ABL gene mutation or amplification. Science.

[6] Shah NP, Nicoll JM, Nagar B, Gorre ME, Paquette RL, Kuriyan J, Sawyers CL (2002). Multiple BCR-ABL kinase domain mutations confer polyclonal resistance to the tyrosine kinase inhibitor imatinib (STI571) in chronic phase and blast crisis chronic myeloid leukemia. Cancer Cell.

[7] Al-Hajj M, Wicha MS, Benito-Hernandez A, Morrison SJ, Clarke MF (2003). Prospective identification of tumorigenic breast cancer cells. Proc Natl Acad Sci U S A.

[8] Pardal R, Clarke MF, Morrison SJ (2003). Applying the principles of stem-cell biology to cancer. Nat Rev Cancer.

[9] Reya T, Morrison SJ, Clarke MF, Weissman IL (2001). Stem cells, cancer, and cancer stem cells. Nature.

[10] Singh SK, Clarke ID, Terasaki M, Bonn VE, Hawkins C, Squire J, Dirks PB (2003). Identification of a cancer stem cell in human brain tumors. Cancer Res.

[11] Jordan CT, Guzman ML, Noble M (2006). Cancer stem cells. N Engl J Med.

[12] Wang J, Ouyang W, Li J, Wei L, Ma Q, Zhang Z, Tong Q, He J, Huang C (2005). Loss of tumor suppressor p53 decreases PTEN expression and enhances signaling pathways leading to activation of activator protein 1 and nuclear factor kappaB induced by UV radiation. Cancer Res.

[13] Rossi DJ, Jamieson CH, Weissman IL (2008). Stems cells and the pathways to aging and cancer. Cell.

[14] Graham SM, Jorgensen HG, Allan E, Pearson C, Alcorn MJ, Richmond L, Holyoake TL (2002). Primitive, quiescent, Philadelphia-positive stem cells from patients with chronic myeloid leukemia are insensitive to STI571 in vitro. Blood.

[15] Bhatia R, Holtz M, Niu N, Gray R, Snyder DS, Sawyers CL, Arber DA, Slovak ML, Forman SJ (2003). Persistence of malignant hematopoietic progenitors in chronic myelogenous leukemia patients in complete cytogenetic remission following imatinib mesylate treatment. Blood.

[16] Cortes J, O'Brien S, Kantarjian H (2004). Discontinuation of imatinib therapy after achieving a molecular response. Blood.

[17] Rousselot P, Huguet F, Rea D, Legros L, Cayuela JM, Maarek O, Blanchet O, Marit G, Gluckman E, Reiffers J, Gardembas M, Mahon FX (2007). Imatinib mesylate discontinuation in patients with chronic myelogenous leukemia in complete molecular remission for more than 2 years. Blood.

[18] Shah NP, Tran C, Lee FY, Chen P, Norris D, Sawyers CL (2004). Overriding imatinib resistance with a novel ABL kinase inhibitor. Science.

[19] Copland M, Hamilton A, Elrick LJ, Baird JW, Allan EK, Jordanides N, Barow M, Mountford JC, Holyoake TL (2006). Dasatinib (BMS-354825) targets an earlier progenitor population than imatinib in primary CML but does not eliminate the quiescent fraction. Blood.

[20] Jorgensen HG, Allan EK, Jordanides NE, Mountford JC, Holyoake TL (2007). Nilotinib exerts equipotent antiproliferative effects to imatinib and does not induce apoptosis in CD34+ CML cells. Blood.

[21] Hu Y, Swerdlow S, Duffy TM, Weinmann R, Lee FY, Li S (2006). Targeting multiple kinase pathways in leukemic progenitors and stem cells is essential for improved treatment of Ph+ leukemia in mice. Proc Natl Acad Sci U S A.

[22] Chen Y, Li D, Li S (2009). The Alox5 gene is a novel therapeutic target in cancer stem cells of chronic myeloid leukemia. Cell Cycle.

[23] Peng C, Chen Y, Yang Z, Zhang H, Osterby L, Rosmarin AG, Li S (2010). PTEN is a tumor suppressor in CML stem cells and BCR-ABL-induced leukemias in mice. Blood.

[24] Li J, Yen C, Liaw D, Podsypanina K, Bose S, Wang SI, Puc J, Miliaresis C, Rodgers L, McCombie R, Bigner SH, Giovanella BC, Ittmann M, Tycko B, Hibshoosh H, Wigler MH, Parsons R (1997). PTEN, a putative protein tyrosine phosphatase gene mutated in human brain, breast, and prostate cancer. Science.

[25] Peiffer SL, Herzog TJ, Tribune DJ, Mutch DG, Gersell DJ, Goodfellow PJ (1995). Allelic loss of sequences from the long arm of chromosome 10 and replication errors in endometrial cancers. Cancer Res.

[26] Gronbaek K, Zeuthen J, Guldberg P, Ralfkiaer E, Hou-Jensen K (1998). Alterations of the MMAC1/PTEN gene in lymphoid malignancies. Blood.

[27] Peng C, Brain J, Hu Y, Goodrich A, Kong L, Grayzel D, Pak R, Read M, Li S (2007). Inhibition of heat shock protein 90 prolongs survival of mice with BCR-ABL-T315I-induced leukemia and suppresses leukemic stem cells. Blood.

[28] Guertin DA, Sabatini DM (2007). Defining the role of mTOR in cancer. Cancer Cell.

[29] Silva A, Yunes JA, Cardoso BA, Martins LR, Jotta PY, Abecasis M, Nowill AE, Leslie NR, Cardoso AA, Barata JT (2008). PTEN posttranslational inactivation and hyperactivation of the PI3K/Akt pathway sustain primary T cell leukemia viability. J Clin Invest.

[30] Larson Gedman A, Chen Q, Kugel Desmoulin S, Ge Y, LaFiura K, Haska CL, Cherian C, Devidas M, Linda SB, Taub JW, Matherly LH (2009). The impact of NOTCH1, FBW7 and PTEN mutations on prognosis and downstream signaling in pediatric T-cell acute lymphoblastic leukemia: a report from the Children's Oncology Group. Leukemia.

[31] Jotta PY, Ganazza MA, Silva A, Viana MB, da Silva MJ, Zambaldi LJ, Barata JT, Brandalise SR, Yunes JA (2010). Negative prognostic impact of PTEN mutation in pediatric T-cell acute lymphoblastic leukemia. Leukemia.

[32] Gutierrez A, Sanda T, Grebliunaite R, Carracedo A, Salmena L, Ahn Y, Dahlberg S, Neuberg D, Moreau LA, Winter SS, Larson R, Zhang J, Protopopov A, Chin L, Pandolfi PP, Silverman LB, Hunger SP, Sallan SE, Look AT (2009). High frequency of PTEN, PI3K, and AKT abnormalities in T-cell acute lymphoblastic leukemia. Blood.

[33] Bruns I, Czibere A, Fischer JC, Roels F, Cadeddu RP, Buest S, Bruennert D, Huenerlituerkoglu AN, Stoecklein NH, Singh R, Zerbini LF, Jager M, Kobbe G, Gattermann N, Kronenwett R, Brors B, Haas R (2009). The hematopoietic stem cell in chronic phase CML is characterized by a transcriptional profile resembling normal myeloid progenitor cells and reflecting loss of quiescence. Leukemia.

[34] de Lavallade H, Finetti P, Carbuccia N, Khorashad JS, Charbonnier A, Foroni L, Apperley JF, Vey N, Bertucci F, Birnbaum D, Mozziconacci MJ A gene expression signature of primary resistance to imatinib in chronic myeloid leukemia. Leuk Res.

[35] Chen ML, Xu PZ, Peng XD, Chen WS, Guzman G, Yang X, Di Cristofano A, Pandolfi PP, Hay N (2006). The deficiency of Akt1 is sufficient to suppress tumor development in Pten+/- mice. Genes Dev.

[36] Chen Y, Hu Y, Zhang H, Peng C, Li S (2009). Loss of the Alox5 gene impairs leukemia stem cells and prevents chronic myeloid leukemia. Nat Genet.

[37] Covey TM, Edes K, Fitzpatrick FA (2007). Akt activation by arachidonic acid metabolism occurs via oxidation and inactivation of PTEN tumor suppressor. Oncogene.

[38] Yilmaz OH, Valdez R, Theisen BK, Guo W, Ferguson DO, Wu H, Morrison SJ (2006). Pten dependence distinguishes haema-topoietic stem cells from leukaemia-initiating cells. Nature.

[39] Zhang J, Grindley JC, Yin T, Jayasinghe S, He XC, Ross JT, Haug JS, Rupp D, Porter-Westpfahl KS, Wiedemann LM, Wu H, Li L (2006). PTEN maintains haematopoietic stem cells and acts in lineage choice and leukaemia prevention. Nature.

[40] Stiles B, Groszer M, Wang S, Jiao J, Wu H (2004). PTENless means more. Dev Biol.

[41] Ito K, Bernardi R, Morotti A, Matsuoka S, Saglio G, Ikeda Y, Rosenblatt J, Avigan DE, Teruya-Feldstein J, Pandolfi PP (2008). PML targeting eradicates quiescent leukaemia-initiating cells. Nature.

[42] Guo W, Lasky JL, Chang CJ, Mosessian S, Lewis X, Xiao Y, Yeh JE, Chen JY, Iruela-Arispe ML, Varella-Garcia M, Wu H (2008). Multi-genetic events collaboratively contribute to Pten-null leukaemia stem-cell formation. Nature.

[43] Hu Y, Chen Y, Douglas L, Li S (2009). beta-Catenin is essential for survival of leukemic stem cells insensitive to kinase inhibition in mice with BCR-ABL-induced chronic myeloid leukemia. Leukemia.

[44] Zhao C, Blum J, Chen A, Kwon HY, Jung SH, Cook JM, Lagoo A, Reya T (2007). Loss of beta-catenin impairs the renewal of normal and CML stem cells in vivo. Cancer Cell.

[45] Wang Y, Krivtsov AV, Sinha AU, North TE, Goessling W, Feng Z, Zon LI, Armstrong SA (2010). The Wnt/beta-catenin pathway is required for the development of leukemia stem cells in AML. Science.

